# TGFBI Expressed by Bone Marrow Niche Cells and Hematopoietic Stem and Progenitor Cells Regulates Hematopoiesis

**DOI:** 10.1089/scd.2018.0124

**Published:** 2018-10-25

**Authors:** Sofieke E. Klamer, Yvonne L. Dorland, Marion Kleijer, Dirk Geerts, William E. Lento, C. Ellen van der Schoot, Marieke von Lindern, Carlijn Voermans

**Affiliations:** ^1^ Sanquin Research and Landsteiner Laboratory, Department of Hematopoiesis, Academic Medical Center, University of Amsterdam, Amsterdam, the Netherlands.; ^2^ Sanquin Research and Landsteiner Laboratory, Department of Molecular and Cellular Hemostasis, Academic Medical Center, University of Amsterdam, Amsterdam, the Netherlands.; ^3^ Department of Medical Biology, Academic Medical Center, University of Amsterdam, Amsterdam, the Netherlands.; ^4^ Department of Pharmacology, Duke University, Durham, North Carolina.; ^5^ Sanquin Research and Landsteiner Laboratory, Department of Experimental Immunohematology, Academic Medical Center, University of Amsterdam, Amsterdam, the Netherlands.; ^6^ Department of Hematology, Academic Medical Center, Amsterdam, the Netherlands.

**Keywords:** TGFBI, CAFC, LTC-CFC, migration, lineage differentiation, ECM

## Abstract

The interactions of hematopoietic stem and progenitor cells (HSPCs) with extracellular matrix (ECM) components and cells from the bone marrow (BM) microenvironment control their homeostasis. Regenerative BM conditions can induce expression of the ECM protein transforming growth factor beta-induced gene H3 (*TGFBI* or *BIGH3*) in murine HSPCs. In this study, we examined how increased or reduced *TGFBI* expression in human HSPCs and BM mesenchymal stromal cells (MSCs) affects HSPC maintenance, differentiation, and migration. HSPCs that overexpressed *TGFBI* showed accelerated megakaryopoiesis, whereas granulocyte differentiation and proliferation of granulocyte, erythrocyte, and monocyte cultures were reduced. In addition, both upregulation and downregulation of *TGFBI* expression impaired HSPC colony-forming capacity of HSPCs. Interestingly, the colony-forming capacity of HSPCs with reduced *TGFBI* levels was increased after long-term co-culture with MSCs, as measured by long-term culture-colony forming cell (LTC-CFC) formation. Moreover, *TGFBI* downregulation in HSPCs resulted in increased cobblestone area-forming cell (CAFC) frequency, a measure for hematopoietic stem cell (HSC) capacity. Concordantly, *TGFBI* upregulation in HSPCs resulted in a decrease of CAFC and LTC-CFC frequency. These results indicate that reduced *TGFBI* levels in HSPCs enhanced HSC maintenance, but only in the presence of MSCs. In addition, reduced levels of *TGFBI* in MSCs affected MSC/HSPC interaction, as observed by an increased migration of HSPCs under the stromal layer. In conclusion, tight regulation of *TGFBI* expression in the BM niche is essential for balanced HSPC proliferation and differentiation.

## Introduction

Hematopoietic stem and progenitor cells (HSPCs) interact with specialized bone marrow (BM) niches, of which mesenchymal stromal cells (MSCs) comprise an essential part. Besides cellular factors, secreted and membrane-bound proteins in these niches are important regulators of HSPCs [1–3]. Comparative gene expression profiling of murine HSPCs in homeostatic and regenerative hematopoietic conditions identified transforming growth factor beta-induced gene H3 (*TGFBI or BIGH3*; NCBI Gene ID 7045) as being upregulated in regenerative conditions, provoked by fluorouracil (5FU) treatment [4].

TGFBI is a secreted extracellular matrix (ECM) protein expressed in various human tissues [5–7], which interacts with various integrin membrane receptors [8,9]. As a component of the ECM, TGFBI associates with collagen, fibronectin, laminin, and glycosaminoglycans [5]. It is implicated in tumorigenesis and metastasis [5,10–12] by regulating cell adhesion and migration [13], and by affecting cell cycle progression through regulation of the microtubule organization [14,15]. We recently showed that enhanced TGFBI expression in HSPCs reduces their adhesion to fibronectin, resulting in a decreased CXCL12-induced migration [16].

While the role of TGFBI in cell-ECM interactions in different tissues is well described [8], little is known about its function in the hematopoietic BM microenvironment. TGFβ, the major activator of *TGFBI* expression, inhibits the proliferation of primitive HSPCs and skews HSPC fate toward myelocytic progenitors [17–21]. This raises the question whether TGFBI has similar effects on hematopoiesis. Interestingly, HSPC adherence to BM-MSCs increased *TGFBI* expression in HSPCs, while also increasing their quiescence [22]. Moreover, *TGFBI* expression is high in murine BM HSPCs compared to fetal liver HSPCs, indicating that TGFBI might become important for HSPCs during migration to and residence in the BM [23]. Furthermore, murine stromal cell lines supportive for HSPCs display elevated *TGFBI* expression levels, and* TGFBI* knockdown zebrafish display severely decreased HSPC numbers, indicating that TGFBI is important for HSC specification [24].

These data suggest that TGFBI plays a key role in shaping the BM microenvironment by regulating HSPC development and localization. The aim of this study is to investigate whether TGFBI expression in human stromal and hematopoietic cells affects human HSPC maintenance and differentiation. Our results indicate that tight regulation of TGFBI expression in both HSPCs and MSCs is essential for a balanced proliferation, differentiation, and homeostasis of human HSPCs.

## Methods

### Human cells

Human material was obtained after informed consent, with approval of the local medical ethics committee (MEC). BM was aspirated from patients undergoing cardiac surgery (permit MEC 04/042, No. 04.17.370; AMC, Amsterdam, The Netherlands), mobilized peripheral blood (MPB) was obtained from leukapheresis material, and cord blood (CB) was collected according to the guidelines of NetCord FACT (by the Sanquin Cord Blood bank, The Netherlands). CD34^+^ cells were selected as described previously [25]. Unless specified otherwise, HSPCs in experiments were CB derived. BM-derived MSCs were isolated and cultured as described previously [26]. L88.5 stromal cells [27] were maintained in Dulbecco's modified Eagle's medium (DMEM) (Lonza; BE12-707F) supplemented with 10% fetal calf serum. For co-cultures, primary MSCs were used as stromal layer, unless indicated differently. See Supplementary Methods for cell culture details (Supplementary Data are available online at www.liebertpub.com/scd).

### Gene and protein detection

Quantitative reverse transcriptase PCR (qRT-PCR), western blot assays, and immunofluorescence imaging were performed as described in Supplementary Data.

### Flow cytometry

Primary (transduced) HSPCs were sorted using an Aria-II cell sorter (Becton-Dickinson, San Jose, CA). For flow cytometry analysis, we used the LSR-II (Becton-Dickinson). To detect TGFBI, cells were fixed in 1% formaldehyde (20 min, 4°C), washed with phosphate-buffered saline containing 0.5% bovine serum albumin and 2 mM ethylenediaminetetraaceticacid, and stained with biotinylated goat polyclonal anti-human TGFBI (R&D Systems) followed by Streptavidin-APC (BD). For total cell staining, cells were incubated in Fix&Perm Cell Permeabilization Kit Medium B (Invitrogen; 10 min at room temperature) after fixation. Antibodies used were as follows: CD34-Pe-Cy7 (8G12), CD38-PerCP (HIT2), CD38-APC (HIT2), CD45RA-FITC (L48), CD45-APC (2D1), CD110-PE (BAH-1), CD41-APC (HIP8), CD15-APC (HI98), CD11b-APC (D12), CD235a-APC (HIR2), CD14-APC (MΦP9; BD), CD14-PerCP-Cy5.5 (M5E2), and CD36-FITC (CLB-IVC7) from BD Biosciences, and CD45-PacificBlue (T29/33; DAKO) and CD71-APC (AC102; Miltenyi). Flow-count fluorospheres were used to quantify cell numbers (Beckman Coulter, Fullerton, CA). Data were analyzed using FacsDiva software (BD) [28,29].

### Lentiviral expression vectors

The pSIN-SFFV-*TGFBI*-IRES-*EGFP* construct was described previously [16]. The pSIN-SFFV-EGFP vector was the corresponding control. *TGFBI*-shRNA vectors (sh2-sh5) or scrambled control shRNA vectors (sh-scr) were derived from the TRCN0000062174 (cgcttgagatcttcaaaca), TRCN0000062175 (ccacatcttgaagtcagcta), TRCN0000062176 (agaaggttattggcactaat), TRCN0000062177 (cctcacctctatgtaccaga), and SHC002 pLKO.1 vectors of the MISSION^®^ shRNA Library (Sigma) and used for virus production and transduction of MSCs followed by puromycin resistance selection. The U6-promoter/shRNA cassettes were cloned into the *Nhe*I site of pRRL156-SIN-EGFP to generate GFP-tagged shRNA constructs. These constructs were used to generate virus as described [16]. Virus titers were determined by transduction of 293T cells in serial virus dilutions, followed by flow cytometry for GFP expression. HSPCs and L88-5 cells were transduced at ∼500 and 100 multiplicity of infection/cell, respectively, and selected by flow cytometry for GFP expression.

### Stem and progenitor assays

CD34^+^ cells were plated at 500 cells/mL in cytokine-supplemented methylcellulose medium (MethoCult H4435; StemCell Technologies, Vancouver, Canada). Cultures were incubated for 12–14 days at 37°C. Colony-forming unit-granulocyte-macrophage (CFU-GM), colonies of at least 40 myelocytic cells, and burst-forming unit-erythrocyte (BFU-E)/colony-forming unit-granulocyte, erythroid, monocyte, macrophage (CFU-GEMM), colonies of at least 200 red cells, were scored by microscopy (Leica, Solms, Germany).

For long-term culture-colony forming cell (LTC-CFC) and cobblestone area-forming cell (CAFC) assays, CD34^+^ cells were plated on confluent monolayers of BM-MSCs, in MyeloCult medium (H5100; StemCell Technologies), supplemented with fresh hydrocortisone (1 μM). Colonies were analyzed according to manufacturer's guidelines. Detailed methods are described in Supplementary Data.

## Results

### *TGFBI* mRNA is expressed in human hematopoietic cells at various levels

**FIG. 1.**
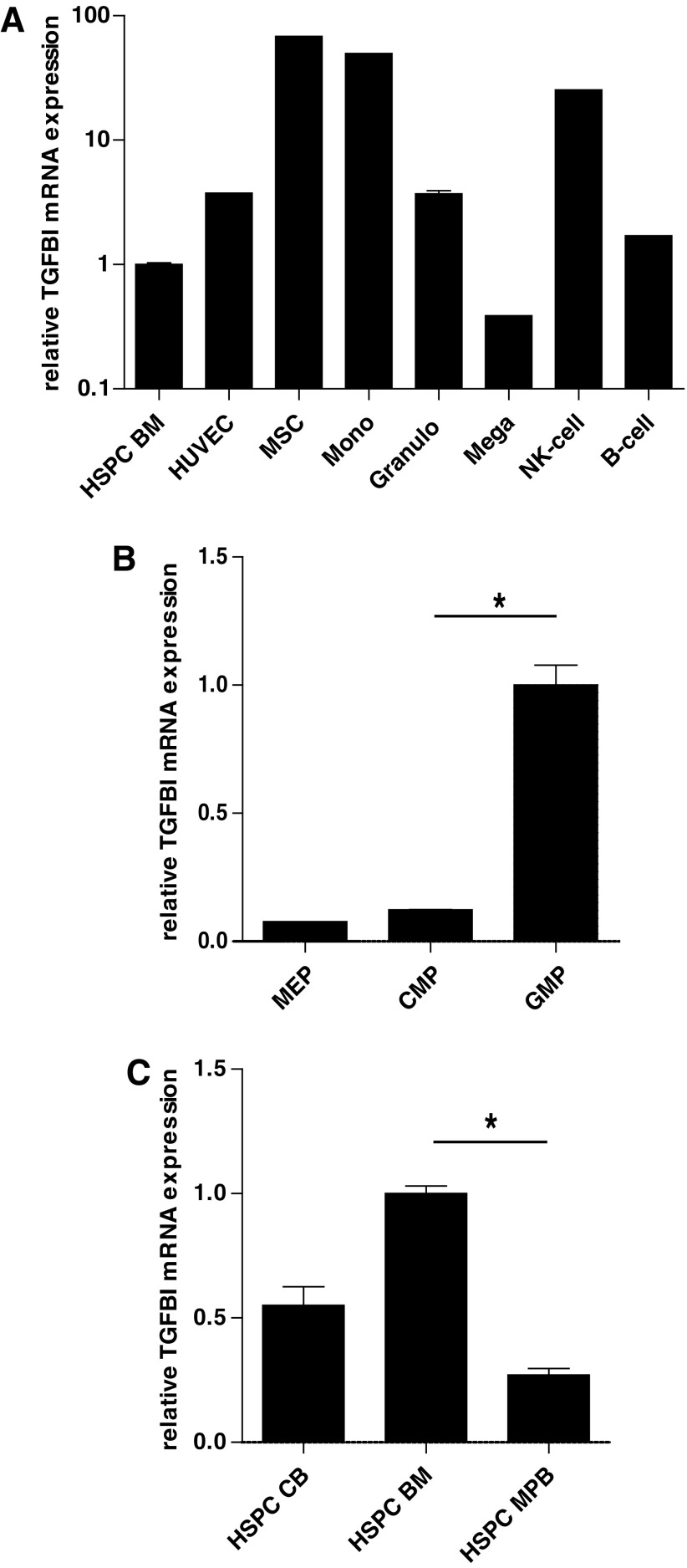
*TGFBI* expression in hematopoietic cells. **(A)** *TGFBI* mRNA expression was determined by qRT-PCR and calculated as fold change relative to GUS reference mRNA expression for different hematopoietic and BM niche cells. *TGFBI* mRNA is higher expressed in monocytes (Mono, *n* = 2) and MSCs (*n* = 2), than in immature CD34^+^ cells (HSPC BM, *n* = 3) and mature megakaryocytes (Mega, *n* = 2). **(B)** *TGFBI* mRNA expression in CD34^+^ subsets of the BM. MACS-isolated CD34^+^ HSPCs were incubated with antibodies and FACS sorted in the following progenitor subsets: the CD45^dim^CD34^+^CD38^+^CD45RA^−^CD110^+^ fraction, representing MEPs (*n* = 2), the CD45^dim^CD34^+^CD38^+^CD45RA^−^CD110^−^ fraction, representing CMPs (*n* = 3), and the CD45^dim^CD34^+^CD38^+^CD45RA^+^CD110^−^ fraction, representing GMPs (*n* = 4). RNA was isolated from sorted cell fractions and *TGFBI* expression relative to reference gene *GUS* expression was analyzed by qRT-PCR. The *TGFBI* expression is significantly higher in GMP fractions compared to CMP fractions. *P < 0.05 **(C)** The relative *TGFBI* mRNA expression in CD34^+^ cells of various origin: isolated from CB (*n* = 4), MPB (*n* = 5), and BM (*n* = 4). The *TGFBI* expression is significantly higher in BM CD34^+^ cells compared to MPB-derived CD34^+^ cells. *P < 0.05. *TGFBI*, transforming growth factor beta-induced gene H3; qRT-PCR, quantitative reverse transcriptase PCR; BM, bone marrow; MSC, mesenchymal stromal cell; HSPC, hematopoietic stem and progenitor cell; MEP, megakaryocyte-erythrocyte progenitor; CMP, common myeloid progenitor; GMP, granulocyte-monocyte progenitor; CB, cord blood; MPB, mobilized peripheral blood; MACS, magnetic-activated cell sorting; FACS, fluorescence activated cell sorting.

We first assessed steady-state *TGFBI* mRNA expression in the various cell types of BM tissue (Supplementary Table S1; Supplementary Data are available online at www.liebertpub.com/scd). Compared to BM-derived CD34^+^ HSPCs, *TGFBI* mRNA was highly expressed in human primary MSCs (almost 100-fold higher) and endothelial cells (almost five-fold higher; Fig. 1A). Concerning the differentiated hematopoietic lineages, the *TGFBI* expression in human primary monocytes and NK-cells was highly increased (>20-fold) compared to BM HSPCs and moderately increased (twofold to threefold) in human primary B cells and granulocytes, but decreased in HSPC-derived megakaryocytes (Fig. 1A). The enhanced *TGFBI* mRNA expression in MSCs compared to HSPCs is in line with transcriptome analyses published by others [30].

*TGFBI* mRNA expression was compared in megakaryocyte-erythrocyte progenitors (CD38^+^/CD110^+^/CD45RA^−^), common myeloid progenitors (CD38^+^/CD110^−^/CD45RA^−^), and granulocyte-monocyte progenitors (GMPs: CD38^+^/CD110^−^/CD45RA^+^) (Fig. 1B). The highest expression was observed in GMPs, corresponding to the high *TGFBI* expression in monocytes. These results correspond with gene expression profiling data of hematopoietic cell types [31]. Analysis of *TGFBI* expression in CD34^+^ HSPCs isolated from adult BM, CB, or MPB revealed the highest expression of *TGFBI* in BM-derived HSPCs and the lowest in MPB-derived HSPCs (Fig. 1C).

*TGFBI* was originally identified as a TGFβ-induced gene in lung carcinoma cells [6]. Culturing HSPCs for 72 h in the presence of TGFβ induced a concentration-dependent increase of *TGFBI* mRNA levels up to 80-fold (Supplementary Fig. S1A). Induction of TGFBI by TGFβ in HSPCs increased both total and cell surface-bound TGFBI protein levels as detected by flow cytometry (Supplementary Fig. S1B). Together, these results indicate that TGFBI expression in HSPCs can be induced by TGFβ and that its expression is extensively regulated during hematopoiesis.

### TGFBI protein is highly expressed in BM stromal cells and secreted in the ECM

**FIG. 2.**
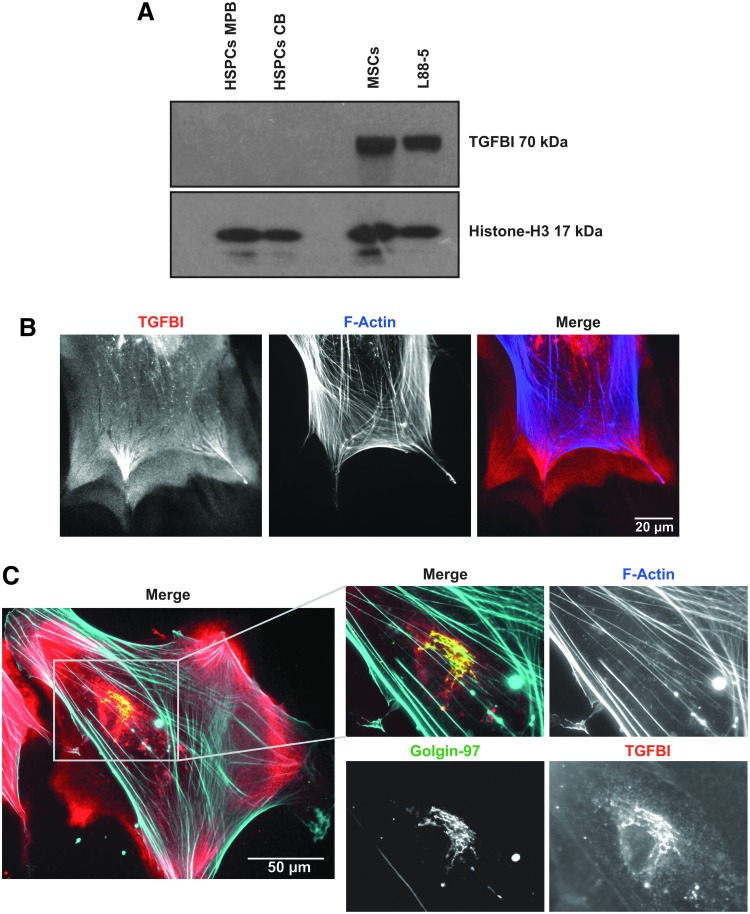
TGFBI expression in BM stromal cells. **(A)** TGFBI protein expression in MPB- and CB-derived HSPCs, primary MSCs, and L88-5 stromal cells was analyzed by western blot. Lysates containing equal cell numbers were loaded and a Histone-H3 antibody (17 kDa) was used as a loading control. TGFBI is detected at 70 kDa. **(B)** Endogenous localization of TGFBI (*red*), co-stained for F-actin (*blue*) in human primary MSCs after 24 h of adhesion to glass. TGFBI is deposited in the extracellular matrix and shows a footprint-like signature when MSCs are migrating. Scalebar indicates 20 μm. **(C)** Endogenous localization of TGFBI (*red*), co-stained for Glogin-97 (*green*) and F-actin (*blue*) in human primary MSCs. Zoomed regions show co-localization (*yellow*) of TGFBI with the golgi-associated protein golgin A1 (golgin-97). Scale bar indicates 50 μm.

TGFBI protein expression in CB HSPCs, MPB HSPCs, MSCs, and L88-5 cells was analyzed on western blot (Fig. 2A). Lysates containing equal cell numbers were loaded and a Histone-H3 antibody was used to confirm equal loading. In line with the mRNA expression levels, HSPCs from different origins express much lower levels of TGFBI compared to stromal cells such as primary MSCs and L88-5 cells. Physiological secretion of TGFBI decreases its protein levels detected in cell lysates and therefore impedes the comparison between mRNA and protein levels for the different cell types. TGFBI is detected on western blot at 70 kDa; however, double bands from proteolytic variants are often detected around 65 kDa (truncated C-terminus) and 60 kDa (truncated N-terminus).

To examine TGFBI secretion by MSCs, we analyzed the localization of TGFBI in MSCs by widefield microscopy. TGFBI protein was abundantly present in the extracellular space, even on noncoated glass surfaces, indicating that TGFBI is indeed secreted and deposited to serve as ECM protein (Fig. 2B). Interestingly, its secretion is most prominently observed at the regions that resemble focal adhesions. Intracellular TGFBI in MSCs is prevalent in vesicular structures, which co-localize with golgin-97, a staining for the Golgi apparatus (Fig. 2C).

### *TGFBI* modifies proliferation of HSPCs

**FIG. 3.**
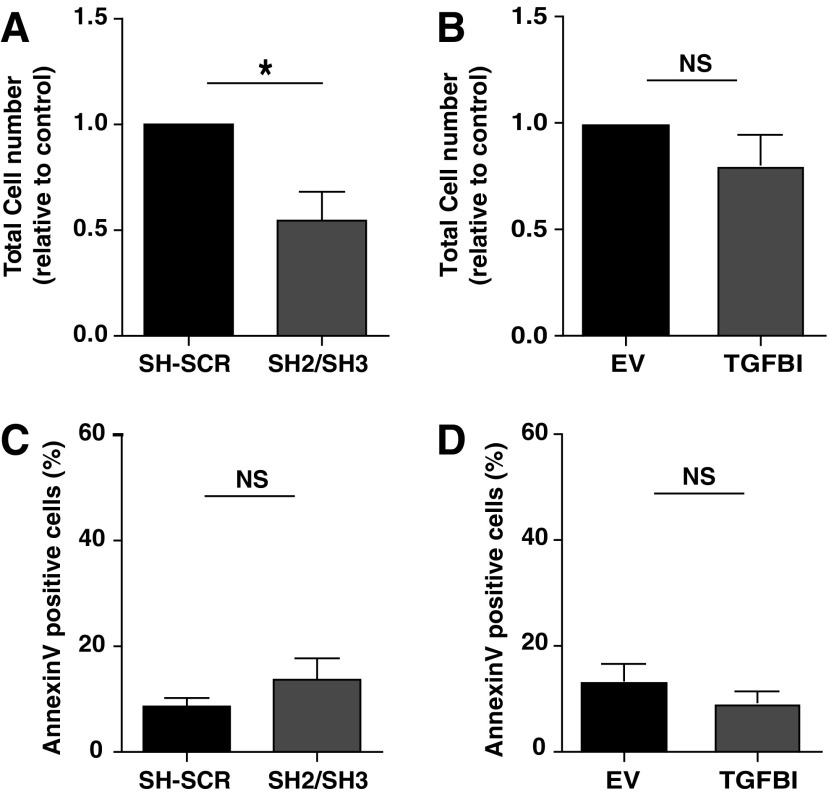
*TGFBI* knockdown in HSPCs reduces total proliferation of HSPCs. HSPCs with *TGFBI* knockdown **(A**, **C)** or *TGFBI* overexpression **(B**, **D)** were cultured in maintenance cocktail and enumerated and analyzed for AnnexinV exposure by flow cytometry 10 days posttransduction. **(A)** HSPCs show decreased cell numbers upon *TGFBI* knockdown (*n* = 5). **(B)** No significant effect on HSPC numbers was observed upon *TGFBI* overexpression (*n* = 4). **(C)** *TGFBI* knockdown (*n* = 5) or **(D)** *TGFBI* overexpression (*n* = 4) did not give a significant effect on AnnexinV exposure in HSPCs. Shown are mean ± SEM. *P < 0.05, NS indicates *P*-value nonsignificant, sh2/sh3 indicates that sh2 or sh3 was used in independent experiments. SCR indicates that a scrambled shRNA control was used. SEM, standard error of the mean.

To investigate the effect of *TGFBI* expression on HSPC expansion, *TGFBI* was upregulated or downregulated in HSPCs that were subsequently cultured for 10 days in maintenance cocktail. The efficiency of knockdown (Supplementary Fig. S2A, B) and overexpression (Supplementary Fig. S2C, D) was verified in HSPCs on mRNA level and protein level. Downregulation of *TGFBI* in HSPCs reduced the expansion in the maintenance culture by 50% (Fig. 3A), while the number of AnnexinV-positive cells was not increased upon *TGFBI* knockdown in HSPCs (Fig. 3C), suggesting that reduced expansion was not due to decreased survival. Overexpression of *TGFBI* did not significantly affect the expansion (Fig. 3B) or the number of AnnexinV-positive cells (Fig. 3D) in the HSPC maintenance culture. *TGFBI* upregulation or downregulation in HSPCs did not induce changes in the phenotypic composition of these cell cultures (Supplementary Fig. S2E, G) or change the percentage of immature HSPCs defined as CD34^+^CD38^−^ (Supplementary Fig. S2F, H) when cultured in a maintenance cocktail.

### *TGFBI* modifies differentiation of HSPCs and proliferation of lineage-committed cells

To investigate the effect of *TGFBI* expression on HSPC lineage commitment and differentiation, CD34^+^-selected HSPCs with upregulated *TGFBI* were cultured in the presence of different cytokine cocktails (See Supplementary Methods for detailed culture conditions) for 14 days and subsequently analyzed for proliferation and specific cell surface markers.

**FIG. 4.**
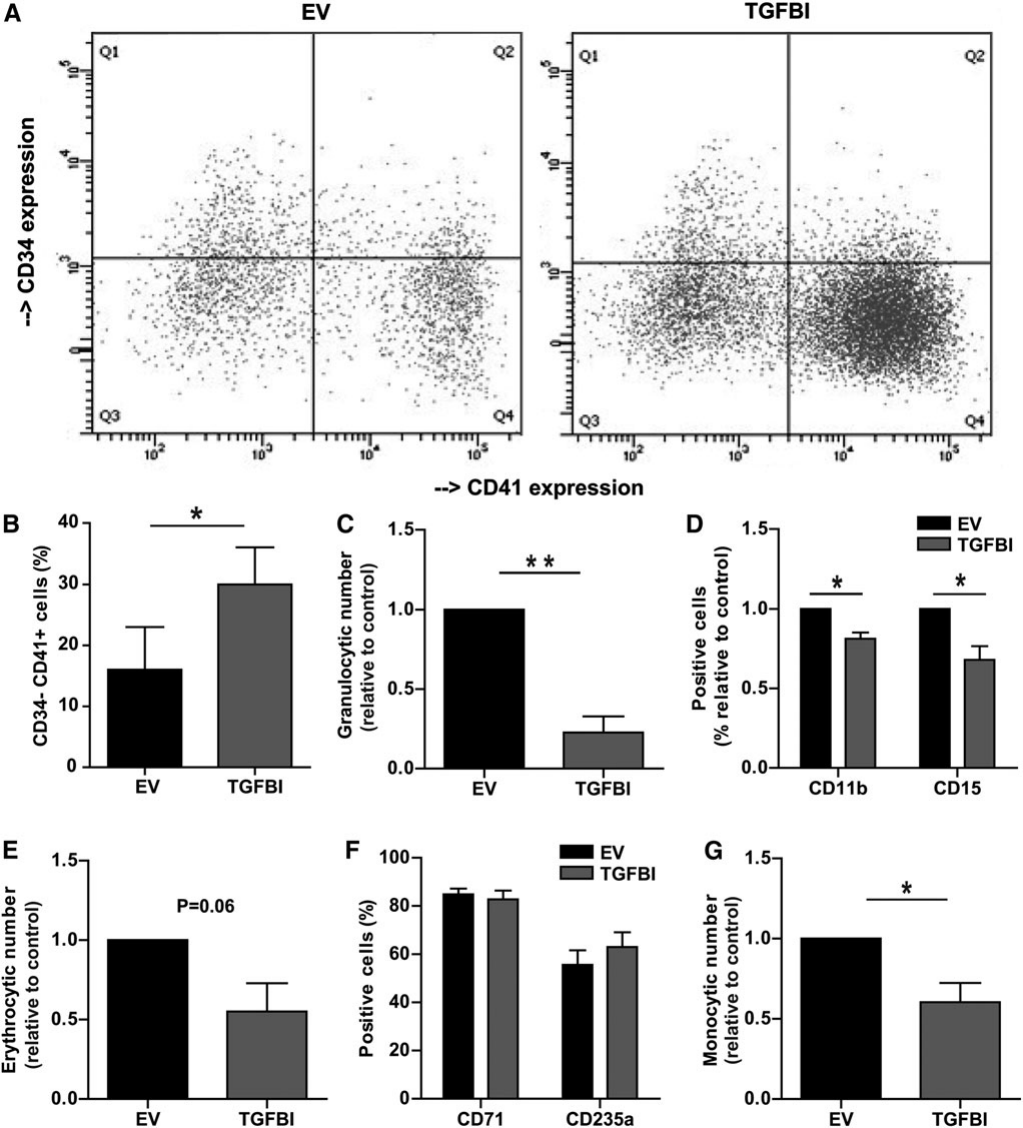
*TGFBI* accelerates megakaryopoiesis and reduces net proliferation in granulocytic, erythrocytic, and monocytic differentiation. HSPCs with *TGFBI* overexpression and control cells were cultured toward different hematopoietic lineages for 14 days, quantified, and analyzed for the expression of specific surface markers by flow cytometry. **(A**, **B)** HSPCs were cultured toward megakaryocytes and analyzed for CD34 and CD41 surface expression. A representative experiment is shown in **(A)**. The percentage of mature megakaryocytes (CD34-CD41^+^) was significantly increased upon *TGFBI* overexpression (*n* = 4). **(C**, **D)** HPSCs were cultured toward granulocytes and analyzed for CD11b and CD15 surface expression. Total granulocytic cell number, and cell surface markers CD11b and CD15 were significantly decreased upon *TGFBI* overexpression (*n* = 3). **(E**, **F)** HSPCs were cultured toward erythroblasts and analyzed for CD71 and CD235a surface expression (*n* = 3). Total erythrocytic cell numbers showed a downward trend upon *TGFBI* overexpression, no significant changes were observed in CD71 and CD235a cell surface expression. **(G)** HSPCs were cultured toward monocytes. Total monocytic cell numbers were significantly decreased upon *TGFBI* overexpression (*n* = 3). Shown are mean ± SEM. *P < 0.05, **P < 0.01, sh2/sh3 indicates that sh2 or sh3 was used in independent experiments.

During differentiation of HSPCs to megakaryocytes, *TGFBI* mRNA expression is downregulated (Fig. 1A). Culturing HSPCs in the presence of thrombopoietin (TPO) and interleukin 1b (IL-1B) results in a loss of CD34 and a gain of CD41 cell surface expression, resembling a megakaryocyte phenotype (Fig. 4A). HSPCs, defined as CD34^+^, overexpressing *TGFBI* and cultured for 14 days in the presence of TPO and IL-1B, showed an increased percentage of CD34^−^CD41^+^ cells (30% ± 7%) compared to control cells (16% ± 6%) (*n* = 4, *P* < 0.05, Fig. 4B). MPB-derived CD34^+^ HSPCs showed a similar trend (data not shown).

Granulocytes can be expanded from CD34^+^ HSPCs by culturing in medium supplemented with stem cell factor (SCF), IL3, IL6, and granulocyte-colony stimulating factor (G-CSF) [32,33]. In these cultures, HSPCs overexpressing *TGFBI* showed a fourfold decrease in proliferation compared to control cultures (*n* = 3, *P* < 0.01, Fig. 4C). During granulocytic differentiation of HSPCs, the cells acquire CD11b and CD15 at the cell surface [34]. *TGFBI* overexpression reduced CD15 expression to 68% ± 8.6% (*n* = 3, *P* < 0.05) and CD11b expression to 81% ± 3.9% (*n* = 3, *P* < 0.05) of control cells (Fig. 4D). This indicated that increased *TGFBI* expression inhibited both proliferation and differentiation of granulocytic cells.

Erythroid differentiation can be identified by high surface expression of transferrin receptor (CD71), followed by expression of glycophorin A (CD235). *TGFBI* overexpression tended to reduce expansion of erythroid cultures to 55% ± 18% (*n* = 3, *P* = 0.06) (Fig. 4E). However, CD71 and CD235 expression did not change significantly upon *TGFBI* overexpression (Fig. 4F), suggesting that *TGFBI* might reduce the net proliferation of erythroblasts, independent of erythroblast differentiation.

Monocytic differentiation is characterized by increased surface CD14 expression, which was not affected by *TGFBI* overexpression (data not shown). At day 14 of the monocytic differentiation culture, HSPCs overexpressing *TGFBI* were decreased to 60% ± 12% compared to control cells (*n* = 3, *P* < 0.05, Fig. 4G).

Thus, the effects of increased *TGFBI* expression depended on the hematopoietic lineage. High levels induced megakaryocytic differentiation and inhibited granulocytic proliferation and differentiation. In the erythroid and monocytic lineage, differentiation was unaffected by *TGFBI*, but proliferation of the culture was reduced.

### Tight regulation of *TGFBI* expression in HSPCs is important for colony-forming capacity

**FIG. 5.**
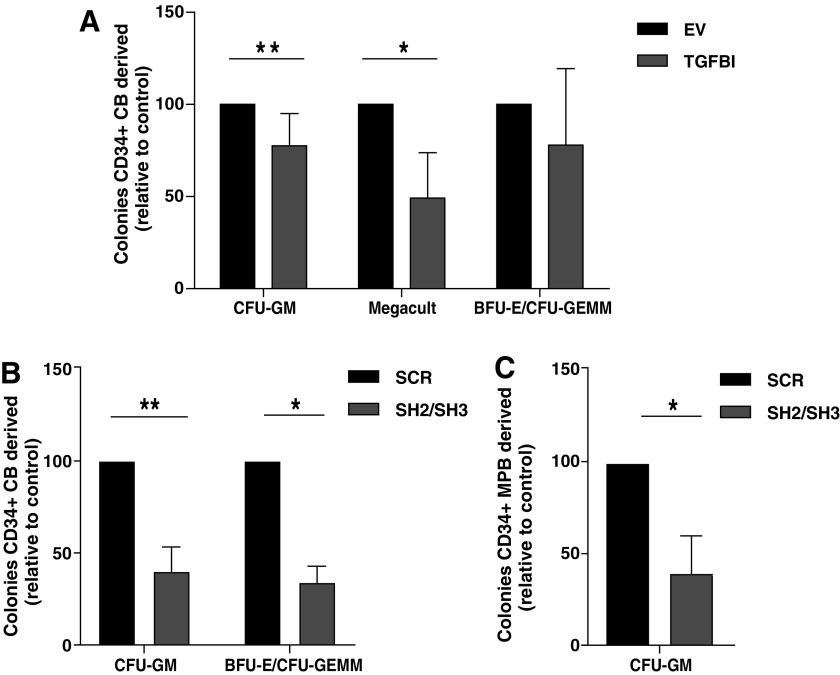
Clonogenic assays with CB or MPB-derived HSPCs with increased *TGFBI* or decreased *TGFBI* expression. **(A)** The number of CFU-GM and BFU-E/CFU-GEMM colonies cultured in methylcellulose medium, and the number of megakaryocyte progenitors cultured in MegaCult produced by CB-derived HSPCs with *TGFBI* overexpression, normalized to controls. Shown are mean ± SEM (*n* = 7 for CFU-GM; *n* = 4 for BFU-E/CFU-GEMM; *n* = 4 for MegaCult). Range of absolute colonies per 500 plated cells for CFU-GM: SCR = 30–100, TGFBI = 20–70, BFU-E/CFU-GEMM: SCR = 20–40, TGFBI = 15–35. **(B**, **C)** *TGFBI* knockdown in CD34^+^ cells derived from CB (*n* = 6) or from MPB (*n* = 4) decreased CFU-GM formation compared to the control. Range of absolute colonies per 500 plated cells for CB: SCR = 25–125, SH2/SH3 = 10–75 and for MPB: SCR = 20–70, SH2/SH3 = 5–45. **(B)** BFU-E/CFU-GEMM formation was also decreased by *TGFBI* knockdown in CD34^+^ cells isolated from CB (*n* = 4). Number of absolute colonies counted per 500 plated cells for CB: SCR = 20–50, SH2/SH3 = 8–15. Colonies were enumerated 12–14 days after plating, each sample was performed in duplicate. *P < 0.05, **P < 0.01, sh2/sh3 indicates that sh2 or sh3 was used in independent experiments. SCR indicates that a scrambled shRNA control was used. CFU-GM, colony-forming unit granulocyte-macrophage; BFU-E, burst-forming unit-erythrocyte; CFU-GEMM, colony-forming unit-granulocyte, erythroid, monocyte, macrophage.

To examine how *TGFBI* expression affects the colony-forming capacity of HSPCs, *TGFBI* was upregulated or downregulated in CD34^+^ HSPCs. Sorted GFP^+^ cells were seeded in MethoCult to assess CFU-GM and BFU-E/CFU-GEMM colonies or in Megacult to assess megakaryocytic colonies. Compared to empty vector controls, *TGFBI* overexpression in CB-derived HSPCs reduced CFU-GM colony formation by 22% (*n* = 4, *P* < 0.01, Fig. 5A) and megakaryocytic colonies by 51% (*n* = 3, *P* < 0.05). No statistically significant decrease in BFU-E/CFU-GEMM colonies was detected, although this result is not clear due to a large variation in colony numbers in the different experiments (*n* = 4, *P* = 0.08).

*TGFBI* knockdown in HSPCs also reduced CFU-GM formation by HSPCs derived from CB by 58% (*n* = 6, *P* < 0.01, Fig. 5B) and MPB by 61% (*n* = 4, *P* < 0.05, Fig. 5C). Furthermore, *TGFBI* knockdown decreased the number of BFU-E/CFU-GEMM by 68% in CB-derived HSPCs (*n* = 4, *P* < 0.05, Fig. 5B).

The small reduction in CFU upon *TGFBI* overexpression and the more pronounced CFU reduction upon *TGFBI* knockdown are in line with the data concerning total proliferation as shown in Fig. 3A and B. Although, the cellular processes perturbed by increased or decreased *TGFBI* expression may be different, these data imply that tight regulation of *TGFBI* expression is essential during hematopoiesis.

### *TGFBI* expression in HSPCs reduces the number of immature hematopoietic cells in co-cultures

**FIG. 6.**
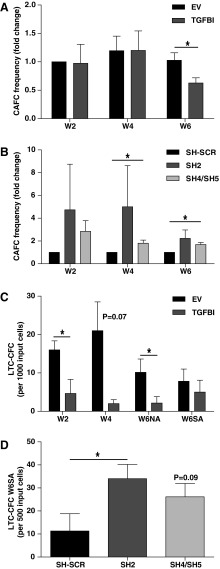
*TGFBI* reduces clonogenic capacity of HSPCs. Clonogenic assays with CB-derived HSPCs with increased *TGFBI* **(A**, **C)** or decreased *TGFBI* expression. **(B**, **D)** CAFC assays **(A**, **B)** show frequency of CAFC in MSC/HSPC co-culture, relative to input cells. LTC-CFC assays **(C**, **D)** show colony-forming capacity of the MSC/HSC co-culture progeny. At indicated time points (week 2, 4, or 6), part of the NA HSPCs or SA HSPCs from MSC/HSPC co-cultures, was plated in methylcellulose medium. LTC-CFC were analyzed after 12–14 days. **(A)** HSPCs overexpressing *TGFBI* in CAFC assays show decreased CAFC frequencies at week 6 of MSC/HSPC co-culture. Shown frequencies are normalized to control conditions at week 2 (*n* = 3). **(B)** HSPCs with *TGFBI* knockdown in CAFC assay show decreased CAFC frequencies at week 4 and 6 of MSC/HSPC co-cultures. Shown frequencies are normalized to control conditions for each week (*n* = 3). **(C)** HSPCs overexpressing *TGFBI* in LTC-CFC assays show reduced colony outgrowth. Shown are mean ± SEM (*n* = 3). **(D)** HSPCs with *TGFBI* knockdown in LTC-CFC show increased colony outgrowth from the stromal adherent HSPC fraction after 6 weeks of MSC/HSPC co-culture (W6SA). Shown are mean ± SEM (*n* = 3). *P < 0.05, sh4/sh5 indicates that sh4 or sh5 was used in independent experiments. CAFC, cobblestone-area-forming cell; LTC-CFC, long-term culture-colony forming cell; HSC, hematopoietic stem cell; NA, nonadherent; SA, stroma adherent.

To investigate whether TGFBI affects maintenance and differentiation of HSPCs in a BM niche environment, we co-cultured HSPCs and primary MSCs. The CAFC assay allows for the quantification of immature HSPCs in a limiting dilution setup. The differentiation and proliferation potential of input HSPCs are measured retrospectively and in the constant presence of MSCs. Results are based on visual endpoints eliminating the effects of medium changes or trypsinization. At week 2 and 4, cobblestone areas represent progenitors and mature hematopoietic cells, whereas cobblestone areas at week 6 represent the more immature HSPCs [2]. *TGFBI* was either upregulated or downregulated in HSPCs and GFP-expressing cells were seeded in CAFC assays. *TGFBI* overexpression in HSPCs did not affect CAFC formation in the first 4 weeks, but at week 6, CAFC frequencies were reduced compared to mock transduced HSPCs (Fig. 6A).

Reduced *TGFBI* expression did not induce a statistically significant change in cobblestone areas at week 2. However, at week 4 and 6, the CAFC frequencies were increased in all individual experiments with reduced *TGFBI* expression. A significant increase was observed with sh4 or sh5 (*n* ≥ 3, *P* < 0.05 at week 4, *P* < 0.01 at week 6), and a similar trend with sh2 (*n* = 3, *P* = 0.19 at week 4 and *P* = 0.12 at week 6, Fig. 6B). Because the efficiency of *TGFBI* knockdown was comparable for the different shRNA constructs and similar responses were observed, data were pooled as indicated. Thus, the number of week-6 CAFCs, which is a measure of the more immature hematopoietic cells, was inversely correlated with *TGFBI* expression. The data suggest that HSPC maintenance was suppressed by increased expression of *TGFBI* and enhanced by reduced *TGFBI* expression.

### *TGFBI* expression in HSPCs decreases hematopoietic progenitor frequencies in co-cultures

Next we investigated how *TGFBI* expression affected progenitor production in LTC-CFC assays. Results of these assays are determined by both HSPC maintenance during culture and colony-forming capacity of progenitors, described in the previous paragraphs. Transduced CD34^+^ cells were co-cultured with primary MSCs and nonadherent (NA) cells derived from the supernatant were seeded in methylcellulose medium after 2 and 4 weeks of co-culture to asses CFCs. After 6 weeks, the complete culture was harvested and both NA- and stroma-adherent (SA) cell fractions were seeded in methylcellulose to determine CFC.

*TGFBI* overexpression reduced the number of CFC in the NA fraction by 70%–90% compared to vector control, displaying 29% at week 2 (*P* < 0.05), 9.5% at week 4 (*P* = 0.07), and 22% at week 6 (*P* < 0.05, Fig. 6C). No difference was observed in the SA fractions at week 6 upon *TGFBI* overexpression.

LTC-CFC capacity of NA HSPCs remained unchanged upon knockdown of *TGFBI* (data not shown). However, the SA HSPCs in which *TGFBI* expression was reduced by the sh2 vector yielded 3.0 ± 2.1-fold more colonies (34.0 ± 6.2 vs. 11.3 ± 7.5, *n* = 3, *P* < 0.01, Fig. 6D). Knockdown of *TGFBI* with vectors sh4 or sh5 resulted in 2.3 ± 1.6-fold more colonies (26.1 ± 5.8 vs. 11.3 ± 7.5, *n* ≥ 3, *P* = 0.09).

The results of the LTC-CFC assay corroborated the results of the CAFC assay. An increased *TGFBI* expression in HSPCs reduced expansion of early progenitors, whereas a decreased *TGFBI* expression in HSPCs increased late CAFC numbers and maintained the production of SA progenitors.

### Reduced TGFBI in MSCs alters HSPC differentiation and increases the migration of HSPCs under the stromal layer

**FIG. 7.**
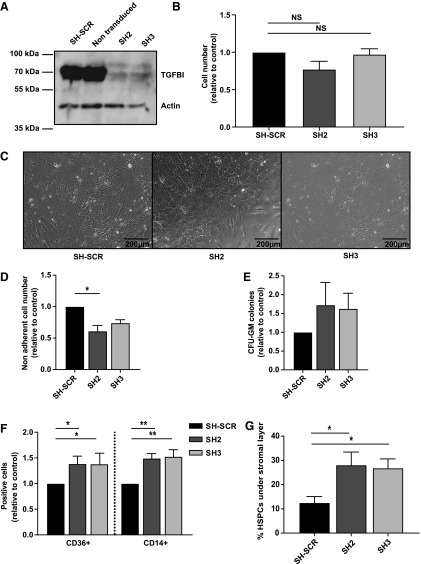
Lack of *TGFBI* in BM-derived MSCs alters HSPC migration. MSCs were transduced with TGFBI-shRNA vectors (sh2 and sh3) or control shRNA vectors (SH-SCR) and replated for co-culture assays with HSPCs. The HSPC compartment was analyzed 1 week after co-culture initiation. **(A)** Western blot analysis of TGFBI knockdown in MSCs, showing efficient knockdown for both shRNA constructs. **(B)** Total MSC cell number, counted 2 weeks posttransduction. *TGFBI* knockdown in MSCs is not affecting proliferation. Shown are normalized mean ± SEM. **(C)** Reduced levels of TGFBI expression did not induce morphological changes as observed by widefield microscopy. MSCs were allowed to adhere for 1 week. A representative experiment is shown. **(D)** Number of total hematopoietic cells as determined by flow cytometry, in co-culture with TGFBI-expressing or nonexpressing MSCs. After 1 week of culture in the absence of TGFBI, a slight decrease in HSPC number is observed (*n* = 3). **(E)** Number of CFU-GM colonies cultured in methylcellulose medium derived from the HSPC compartment from co-culture experiments with MSCs. Presence or absence of TGFBI did not result in significant changes in colony outgrowth. Shown are mean ± SEM (*n* = 3), normalized to control, samples were performed in duplicate. **(F)** NA HSPCs from co-culture with MSCs were analyzed by flow cytometry for surface expression of CD14 and CD36. The percentage of both CD14 (monocyte marker) and CD36 (macrophage marker) was increased after 1 week of co-culture with TGFBI knockdown MSCs (*n* = 3). **(G)** HSPC/MSC co-cultures were live imaged and the number of HSPCs present under the stroma was determined as a percentage of total HSPCs per field of view. Shown are mean ± SEM (*n* = 3), normalized to control, samples were measured in quadruplicate. In a co-culture with MSCs lacking TGFBI expression, an increased number of HSPCs moves underneath the stromal layer compared to a co-culture with TGFBI-expressing MSCs.

TGFBI expressed and secreted by MSCs might affect hematopoiesis in co-culture experiments. To investigate this, we downregulated *TGFBI* in MSCs with two different shRNA constructs (Fig. 7A) and co-cultured these cells together with HSPCs. Efficient TGFBI knockdown in primary MSCs did not affect proliferation (Fig. 7B) or cell morphology (Fig. 7C). Multilineage differentiation capacity of MSCs toward adipocytes or osteoblasts was also not affected by TGFBI knockdown (Supplementary Fig. S3A–D).

In co-culture experiments, a reduction of TGFBI in MSCs by sh2 resulted in a significantly decreased output of NA hematopoietic cells, while a similar trend was observed with sh3 (Fig. 7D). To examine whether reduced TGFBI levels in co-cultures had an effect on the colony-forming capacity of hematopoietic progenitors, the NA cells were seeded in methylcellulose medium after 1 week of co-culture. When CFU-GM colonies were counted after 12–14 days of culture, no differences in colony number was detected (Fig. 7E).

To investigate whether TGFBI expression by MSCs can direct lineage commitment of HSPCs, different surface markers for the myeloid lineage were assessed by flow cytometry (Fig. 7F). Both CD14 and CD36 were significantly upregulated after 1 week of co-culture with MSCs expressing reduced levels of TGFBI, indicating that monocyte and macrophage maturation are enhanced. In addition to co-cultures of TGFBI knockdown MSCs with HSPCs, we also performed co-cultures with TGFBI knockdown L88-5 stromal cells to assess the number of mature and more immature hematopoietic cells. However, these co-cultures did not affect the CAFC capacity of HSPCs (Supplementary Fig. S4A–C).

Since TGFBI is part of the ECM, and is known to decrease adhesion in HSPCs [16], we investigated whether HSPC migration patterns are also altered in our co-culture assays. To evaluate HSPC migration, co-cultures were live imaged through differential interference contrast microscopy and the number of HSPCs migrating under the mesenchymal stromal layer was quantified after 1 week of co-culture (Fig. 7G and Supplementary Movie M1). In these experiments, reduced levels of TGFBI in MSCs resulted in an increased adhesion of HSPCs under the MSC stromal layer. These results corroborate with the HSPC count of the NA fraction (Fig. 7D) and emphasize the importance of TGFBI regulation for the MSC-HSPC interaction [16].

## Discussion

Environmental factors that control maintenance and differentiation of HSC include the ECM protein TGFBI. In this study, we investigated the role of TGFBI in stromal and hematopoietic cells, in particular, its role in HSPC maintenance and differentiation. We demonstrate that *TGFBI* expression maintains HSPC immaturity, as shown by a decrease in late-cobblestone area formation (late-CAFC) in *TGFBI*-overexpressing HSPCs and an increase in CAFC in *TGFBI* knockdown HSPCs. Enhanced *TGFBI* expression impaired the colony-forming capacity of HSPCs in the absence of stromal support, and in the presence of MSCs, late-CAFC, a measure for HSPC immaturity, was reduced. *TGFBI* knockdown in HSPCs enhanced late-CAFC and adherent LTC-CFC, indicating HSC maintenance. Furthermore, TGFBI enhanced megakaryocytic differentiation, while it reduced expansion during monocytic, granulocytic, and erythrocytic differentiation.

Knockdown of *TGFBI* in MSCs has no notable effect on culture-expanded MSCs themselves; however, their hematopoietic support alters, resulting in an increased monocyte/macrophage outgrowth and an increased migration of HSPCs under the stromal cell layer. We conclude that, tight regulation of *TGFBI* expression in both HSPCs and stromal cells is essential for normal proliferation, differentiation, and homeostasis of HSPCs.

### *TGFBI* controls HSPCs in the context of supporting MSCs

In the hematopoietic BM niche, TGFβ and TGFBI contribute to microniches that regulate hematopoiesis [35]. Compared to HSPCs, MSCs express and secrete more TGFBI [8,24,36,37], but modulation of *TGFBI* expression in HSPCs themselves controls their maintenance, even in co-cultures with MSCs.

Several observations support the conclusion that TGFBI mediates its effect by both cell intrinsic and paracrine mechanisms. Our data clearly show an intrinsic effect of TGFBI through its knockdown and overexpression in HSPCs. Colony-forming capacity of HSPCs in semisolid colony assays is decreased by both, knockdown and overexpression of *TGFBI*, indicating that the intrinsic pathways depend on a tight balance of *TGFBI* expression. Moreover, even in the presence of MSCs that secrete high levels of TGFBI, modulation of the intrinsic *TGFBI* expression in HSPCs controls their functional outcome as shown by the CAFC and LTC-CFC assays, supporting the idea that TGFBI acts cell autonomously in HSPCs. The paracrine effect of TGFBI is observed in co-cultures; decreased TGFBI expression in MSCs results in decreased total hematopoietic proliferation and a relative increase in hematopoietic cells skewed toward monocyte macrophage differentiation.

The TGFBI paracrine effect might also be attributed to changes in HSPC adhesion in these co-cultures. We previously showed that HSPCs bind TGFBI through integrins on their cell membrane. *TGFBI* overexpression in HSPCs reduced their binding to fibronectin by competitive binding of integrins [9,16]. In this study, we showed that TGFBI can be detected both intracellular and on the cell surface of both HSPCs and MSCs. The interaction of HSPCs with MSCs is dynamic and crucial for HSC maintenance [2,38]. HSPCs that express low TGFBI levels may readily bind to the ECM produced by MSCs, whereas increased TGFBI production by HSPCs may block this interaction and reduce HSPC-MSC interactions. The same mechanism may explain the observed increase of HSPC-MSC interaction in co-cultures of MSCs expressing lower levels of TGFBI; adhesion molecules secreted by the MSCs at the substrate surface are more accessible for HSPC integrins in the absence of competitively secreted TGBFI from the MSC (Fig. 7G). Together, this implies that TGFBI modifies intracellular signals of HSPCs and alters the interaction with MSCs. Both mechanisms may contribute to the functional outcome of the HSPCs.

### *TGFBI* during homeostatic and regenerative hematopoiesis in the BM niche

*TGFBI* is one of many TGFβ target genes in HSPCs. In vitro, TGFβ is known to inhibit HSPC proliferation and promote HSPC quiescence [17,20,39]. In vivo studies show similar results in regenerative conditions [18,40,41], although in homeostatic conditions, HSPCs can remain quiescent, even at low levels of TGFβ, indicating that the context of TGFβ signaling in HSPCs determines its effect [35,40].

In our experiments, *TGFBI* downregulation in HSPCs increased the maintenance of immature HSPCs compared to control HSPCs in the context of stromal cells.

This opposite effect compared to TGFβ signaling might be because upregulation of TGFBI in HSPCs negatively regulates the other signaling pathways of TGFβ that initiate HSPC quiescence, explaining the differences in CFU outgrowth in the presence or absence of stroma. TGFβ is not the only factor inducing *TGFBI* expression. *TGFBI* is also upregulated in murine HSPCs following HSPC mobilization by G-CSF [42], or after 5FU treatment [4]. In these regenerative conditions, active TGFβ is initially reduced [40,43], possibly related to hematopoietic recovery, while it is upregulated at later time points, possibly to induce quiescence in HSPCs again [40]. Lineage-committed progenitors are less responsive to TGFβ and expand during hematopoietic recovery, while HSCs should be maintained. We showed that high *TGFBI* levels decrease colony formation in vitro, which may represent increased HSPC differentiation in regenerative conditions.

*TGFBI* expression in HSPCs depends on the source of the cells. We detected the highest *TGFBI* expression in BM-derived HSPCs, compared to other sources of HSPCs. *TGFBI* is upregulated in HSPCs upon binding to MSCs [25], and only BM-derived HSPCs are in close contact with MSCs. MPB-derived HSPCs, on the other hand, are mobilized by G-CSF that increases *TGFBI* expression in BM-derived HSPCs [42]. Nevertheless, the G-CSF-mobilized HSPCs isolated from peripheral blood showed lower *TGFBI* mRNA expression compared to BM-derived HSPCs. The higher *TGFBI* expression in BM-derived HSPCs may also be correlated with the increased percentages of active cycling HSPCs in BM compared to MPB [25], suggesting that *TGFBI* expression is more pronounced in HSPC populations with a higher mitotic activity.

### *TGFBI* regulates hematopoietic lineages differentially

We found that megakaryocytic differentiation was enhanced upon *TGFBI* overexpression, whereas granulocytic differentiation was inhibited. Thus, the effect of enhanced *TGFBI* expression depends on the hematopoietic lineage. Increased maturation of megakaryocytic cells upon enhanced *TGFBI* expression is also in line with the recently identified critical role of TGFBI in platelet activation and function [44]. Altogether our data suggest that tight regulation of human *TGFBI* levels in HSPCs and MSCs is essential for normal proliferation, differentiation, and homeostasis of HSPCs.

## Supplementary Material

Supplemental data

Supplemental data
