# Supplementary material for: TGFBI Expressed by Bone Marrow Niche Cells and Hematopoietic Stem and Progenitor Cells Regulates Hematopoiesis
Source: Stem Cells Dev. 2018 Oct 25;27(21):1494–506. doi: 10.1089/scd.2018.0124 (PMC6209430; doi:10.1089/scd.2018.0124)
Supplement: Supplemental data [file Supp_Data.pdf]

Supplementary Data

Supplementary Methods

Human cell cultures

To expand hematopoietic stem and progenitor cells (HSPCs), $CD34^+$ cells were cultured in StemSpan (StemCell Technologies, Grenoble, France) cocktail 1: thrombopoietin (TPO, 10 ng/mL, PeliKine M32305; Sanquin, Amsterdam, the Netherlands), stem cell factor (SCF) (100 ng/mL, 255-SC; R&D Systems, Minneapolis, MN), FLT3L (100 ng/mL, 130-093-854; Miltenyi), interleukin (IL)3 (20 ng/mL, 200-03; Peprotech, Rocky Hill, NJ), and IL6 (20 ng/mL, 130-093-931; Miltenyi) [S1,S2]. Cultures were shifted to maintenance cocktail 2 (cocktail 1 minus SCF) on day 3.

Megakaryocytic cultures were initialized in CellGro (CellGenix, Freiburg, Germany) supplemented with cocktail 2, shifted to TPO (100 ng/mL) and IL-1β (20 ng/mL) on day 3, and diluted with TPO (100 ng/mL) and IL6 (20 ng/mL) on day 10.

Monocytic differentiation was initialized in StemSpan medium, containing SCF (50 ng/mL), FLT3L (50 ng/mL), and IL3 (10 ng/mL), changed on day 3 to Iscove's modified Dulbecco's medium (IMDM) (Lonza, Cologne, Germany) with 10% special fetal calf serum (FCS; HyClone, Thermo Scientific, Breda, The Netherlands), SCF (50 ng/mL), FLT3L (100 ng/mL), IL3 (20 ng/mL), IL6 (20 ng/mL), granulocyte macrophage colony-stimulating factor (GM-CSF) (20 ng/mL), and macrophage colony-stimulating factor (M-CSF) (50 ng/mL), diluted in IMDM + FCS, M-CSF (50 ng/mL), GM-CSF (20 ng/mL), and IL3 (20 ng/mL) on day 7, and cultured in M-CSF (50 ng/mL) from day 10.

Granulocytic cultures started similar to monocytic cultures, except that G-CSF (30 ng/mL) replaced IL6 on day 3. Cultures were diluted in IMDM + FCS, including IL3 (10 ng/mL) and G-CSF (30 ng/mL), on day 6, with G-CSF (30 ng/mL) from day 10.

Erythrocytic cultures were initialized in StemSpan plus maintenance cocktail 1; on day 3; erythropoietin (EPO) (2 U/mL), SCF (100 ng/mL), and dexamethasone (1 μM; Sigma) were added. Transforming growth factor beta (TGFβ, 0.2–20 ng/mL; BD, Franklin Lakes, NJ) was added to HSPC cultures when indicated. TPO (100 ng/mL) was replaced by Nplate (10 ng/mL; Amgen, Thousand Oaks, CA), when indicated.

Bone marrow (BM)-derived mesenchymal stromal cells (MSCs) were isolated and cultured as described previously [S3]. L88.5 stromal cells were cultured in Dulbecco's modified Eagle's medium (DMEM) (Lonza; BE12-707F) supplemented with 10% FCS. For co-cultures, MSCs passage 3–4 cultured in DMEM (Lonza; BE12-707F) were transduced with transforming growth factor beta-induced gene H3 (*TGFBI*)-shRNA vectors (Sh2 or Sh3) or control shRNA vectors (Sh-scr) for 4 days and after puromycin selection of 3 days, seeded into 24-well plates, 5×10^4 cells/well. After 1 day, MSCs were irradiated with 12 Gy and 1 day later, DMEM was replaced with 500 μL MyeloCult medium (H5100; StemCell Technologies) containing 5×10^3 HSPCs. Half the medium was refreshed weekly and nonadherent fractions were analyzed 1 and 2 weeks after starting co-culture.

Quantitative reverse transcriptase PCR

RNA was extracted with an RNeasy Mini Kit (74106; Qiagen, Hilden, Germany), concentration measured by Nanodrop bioanalyzer. cDNA was synthesized with random primers and M-MLV Reverse Transcriptase (58875 and 28025-021; Invitrogen, Carlsbad, CA). Quantitative real-time PCR was performed on a StepOne Plus (Applied Biosystems, Warrington, United Kingdom). Primers and probes used are listed in Supplementary Table S1. Oligonucleotide probes

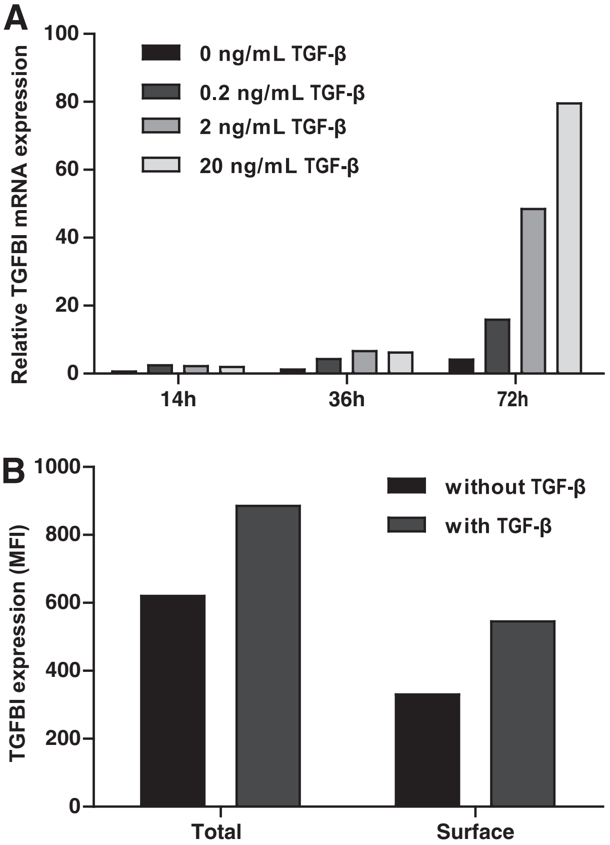

SUPPLEMENTARY FIG. S1. TGFβ induces TGFBI mRNA and protein expression in HSPCs. HSPCs were cultured in the presence of maintenance cytokine cocktail [SCF, Flt3L, interleukin (IL)6, IL3, and thrombopoietin] and various concentrations of recombinant TGFβ. **(A)** RNA was isolated at indicated time points and *TGFBI* expression was analyzed. *GUS* was analyzed to normalize the *TGFBI* expression, and fold changes were calculated relative to the expression at time point zero. A representative experiment is shown ($n=3$). **(B)** TGFBI protein expression in HSPCs is induced by TGFβ treatment, and analyzed by flow cytometry after 5 days of culture. To distinguish between total TGFBI levels and cell surface TGFBI protein levels, cells were either permeabilized or nonpermeabilized before IF staining. Mean fluorescence intensity of TGFBI staining is depicted. A representative experiment is shown ($n=3$). *TGFBI*, transforming growth factor beta-induced gene H3; HSPC, hematopoietic stem and progenitor cell; IF, immunofluorescence; SCF, stem cell factor.

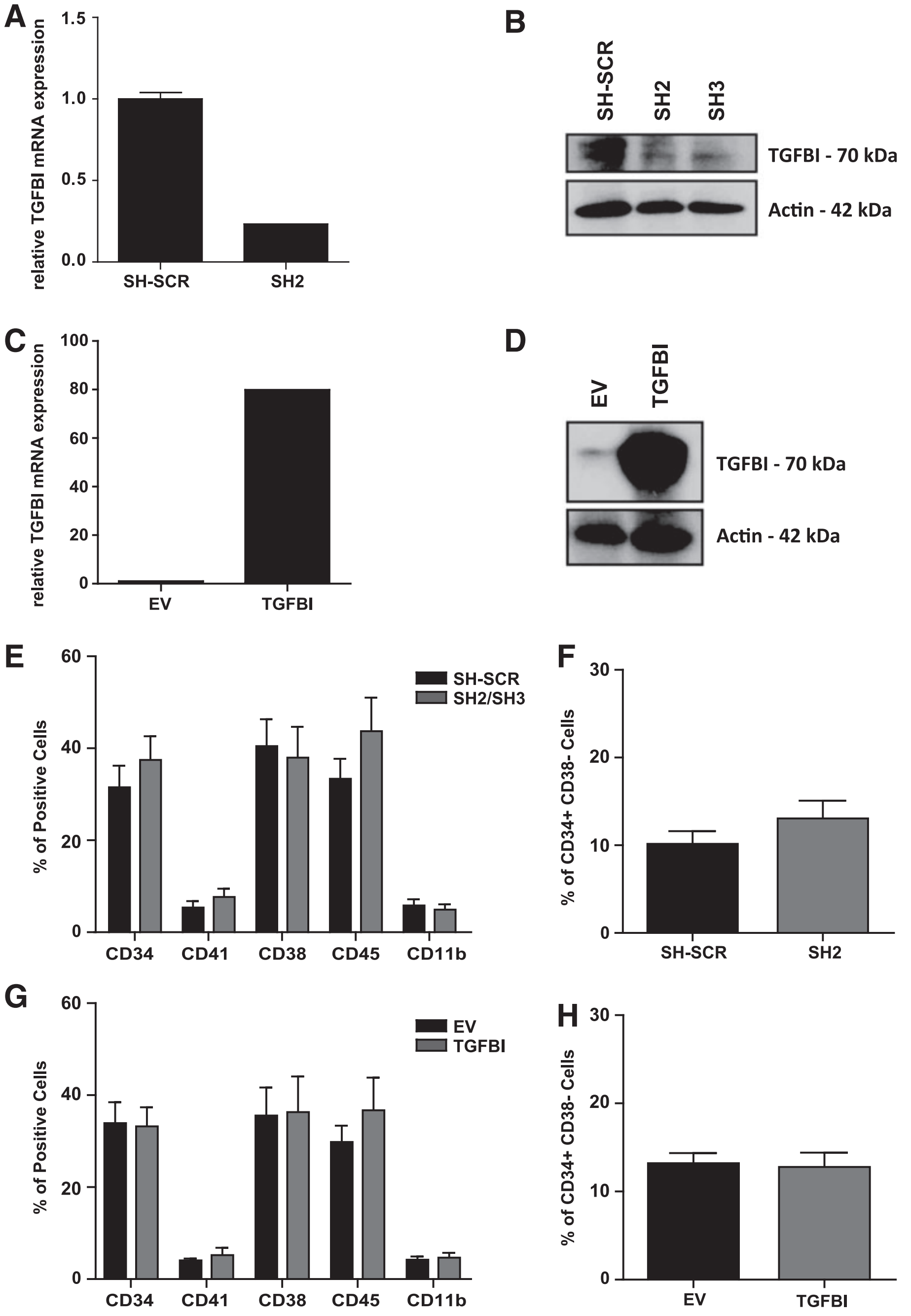

SUPPLEMENTARY FIG. S2. Effective overexpression and *TGFBI* knockdown in HSPCs. **(A)** Analysis of *TGFBI* mRNA from CB-derived HSPCs with *TGFBI* knockdown, relative to control, 2 days after transduction and immediately after FACS for GFP$^+$ cells. Shown are mean ± standard deviation ($n=2$). **(B)** Western blot analysis of TGFBI in CB-derived HSPCs with TGFBI knockdown, 4 days after transduction. A representative experiment is shown ($n=3$). **(C)** Analysis of *TGFBI* mRNA in mobilized peripheral blood-derived HSPCs with *TGFBI* overexpression, 2 days after transduction and immediately after FACS for GFP$^+$ cells. A representative experiment is shown ($n=2$). **(D)** Western blot analysis of TGFBI in CB-derived HSPCs with TGFBI overexpression, 4 days after transduction. A representative experiment is shown ($n=3$). **(E, F)** Phenotypic analysis of HSPCs with TGFBI knockdown 10 days posttransduction, cultured in maintenance cocktail. Graphs show the percentage of total cells stained positive for **(E)** CD34, CD41, CD38, CD45, and CD11b and **(F)** the percentage of immature HSPCs defined as CD34$^+$CD38$^-$ ($n=4$). **(G, H)** Phenotypic analysis of HSPCs with TGFBI overexpression 10 days posttransduction, cultured in maintenance cocktail. Graphs show the percentage of total cells stained positive for **(E)** CD34, CD41, CD38, CD45, and CD11b and **(F)** the percentage of immature HSPCs defined as CD34$^+$CD38$^-$ ($n=4$). CB, cord blood; FACS, fluorescence activated cell sorting.

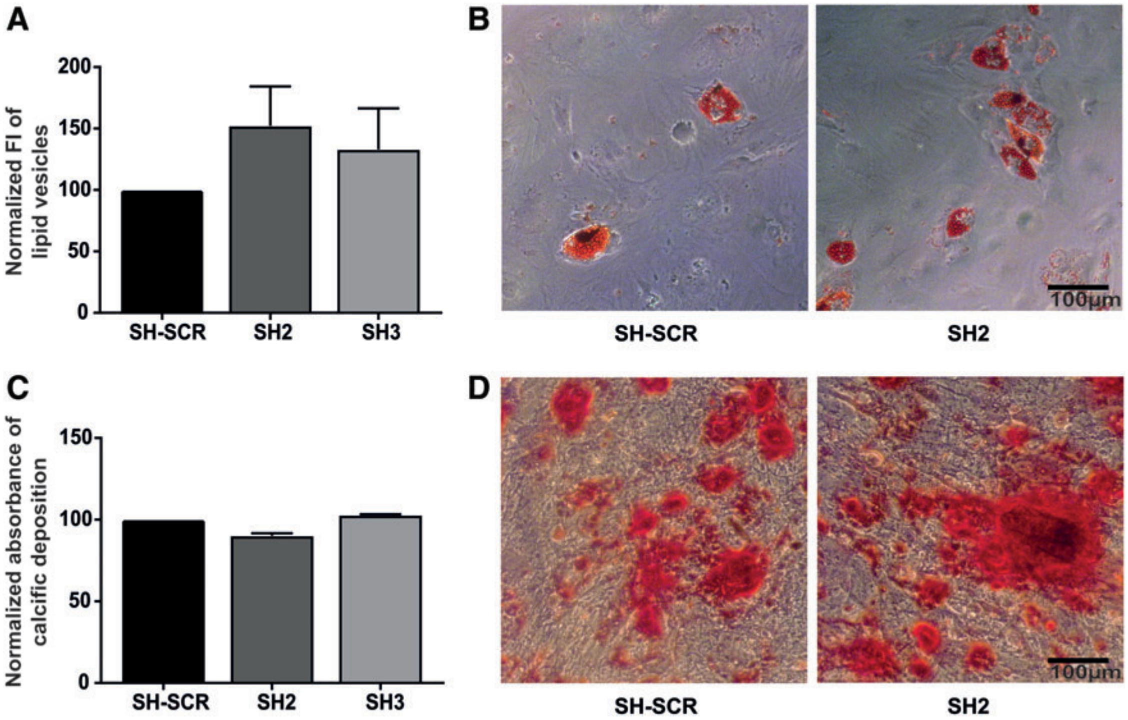

SUPPLEMENTARY FIG. S3. Effective *TGFBI* knockdown in MSCs is not affecting differentiation potential of MSCs. MSC passage 3–4 were cultured in the presence of adipocyte or osteoblast differentiation media for 3 weeks and subsequently analyzed for lipid or calcific deposition. Differentiation potential toward adipocytes **(A, B)** and osteoblasts **(C, D)** is not changed in TGFBI knockdown MSCs compared to control MSCs. Shown are mean ± standard error of the mean ($n = 3$). MSC, mesenchymal stromal cell.

were used for detection of *TGFBI* and *GUS*; Sybrgreen dye (Sybrgreen mastermix; Applied Biosystems) was used for other amplifications. The mRNA expression was calculated as fold change relative to the *GUS* mRNA expression ($2^{-\Delta Ct}$).

Cobblestone-area-forming-cell

For cobblestone area-forming cell (CAFC) assays, $CD34^+$ cells were plated in serial dilutions of two 1,000 cells per well (96 wells plate) on confluent monolayers of BM MSCs.

Cells were cultured in MyeloCult medium (H5100; StemCell Technologies), supplemented with fresh hydrocortisone (1 µM), half the medium was refreshed weekly, and cobblestone areas were counted after 2, 4, and 6 weeks of MSC/HSPC co-culture according to manufacturer's guidelines. Cobblestone areas from CAFC assays were defined as ≥5 phase-dark, closely associated cells. The proportion of CAFC relative to input cells was calculated using limited dilution software (ELDA: http://bioinf.wehi.edu.au/software/elda/)

Long-term culture-colony forming cell

Long-term culture-colony forming cell (LTC-CFC) assays were performed in parallel to CAFC assays to analyze the colony-forming capacities of the CAFC progeny.

For the LTC-CFC assay, 1,000–3,000 $CD34^+$ cells per well (six-well plate) were plated on confluent monolayers of BM MSCs, in MyeloCult medium (H5100; StemCell Technologies), supplemented with fresh hydrocortisone (1 µM).

At indicated time points (week 2, 4, or 6), part of the co-culture-supernatant, containing nonadherent HSPCs, was plated in methylcellulose medium (MethoCult H4435; StemCell Technologies, Vancouver, Canada). The stromal MSC layer, including stromal adherent HSPCs, was harvested at week 6 and plated on methylcellulose medium. Each sample was plated in duplicate and colonies were counted 12–14 days after plating.

Western blotting

Equal cell numbers were lysed and equal lysate volumes were used for western blot analysis, as described previously [S4]. Histone-H3 or Actin was used to confirm equal loading. Primary antibodies were as follows: goat polyclonal anti-human TGFBI (R&D Systems), mouse monoclonal anti-human ACTIN (clone AC-40; Sigma, MO), and rabbit polyclonal anti-human Histone H3 (Santa Cruz; SC-10809). Secondary antibodies were as follows: goat-anti-mouse immunoglobulin G (IgG)-horseradish peroxidase (HRP; DAKO, Glostrup, Denmark), mouse-anti-rabbit IgG-HRP (Southern Biotech, Birmingham, AL), or HRP-conjugated streptavidin (Thermo Scientific).

Immunofluorescent microscopy

Cells were cultured on fibronectin (Sanquin)-coated coverslips, fixed (10 min) in 4% paraformaldehyde in phosphate-buffered saline (PBS) (supplemented with 1 mM $CaCl_2$ and 0.5 mM $MgCl_2$), permeabilized with 0.5% Triton X100 in PBS (5 min), and blocked with 2% bovine serum

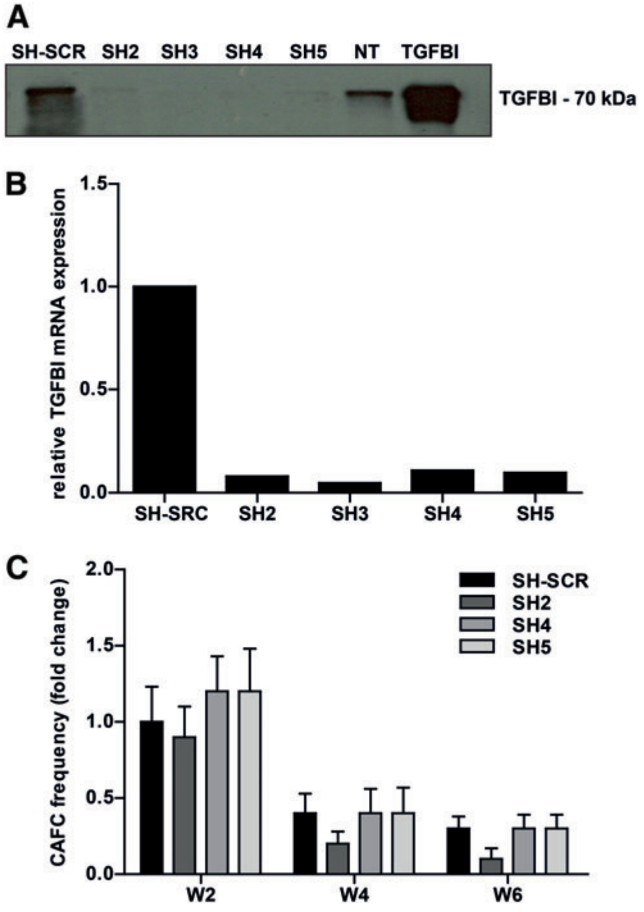

SUPPLEMENTARY FIG. S4. Effective *TGFBI* knockdown in L88-5 stromal cells does not reduce CAFC formation in co-culture assays with HSPCs. **(A)** TGFBI protein expression in L88.5 stromal cells. Samples show TGFBI knockdown (sh2–sh5), control cells (sh-SCR), nontransduced cells (NT), and cells with overexpression (TGFBI). A representative experiment is shown ($n=3$). **(B)** *TGFBI* mRNA expression in L88.5 *TGFBI* knockdown (sh2–sh5) or control cells (sh-SCR). The *TGFBI* expression was normalized to levels in control cells. A representative experiment is shown ($n=3$). **(C)** L88.5 stromal cells with *TGFBI* knockdown (sh2–sh5) or control cells (sh-SCR), co-cultured with CB-derived HSPCs in CAFC assays. The frequency of CAFC relative to input cells was calculated using limited dilution software (ELDA) and normalized to that in the vector control condition at week 2 ($n=1$). CAFC, cobblestone area-forming cell.

albumin (30 min) in PBS. Coverslips were stained with the following antibodies: Goat polyclonal anti-human TGFBI (R&D Systems; AF2935), Chicken anti-Goat AF594 (Invitrogen; A21468), Phalloidin PromoFluor-415 (PromoKine; PK-PF415-7-01) (Fig. 1E), Golgin-97 (Invitrogen; A21270), and Chicken anti-mouse AF488 (Invitrogen; A21200), mounted in Mowiol4-88/DABCO solution and imaged using inverted Zeiss widefield Axiovert 200 with a 63× and 40× objective, respectively. For live-cell imaging, co-cultures in 24-well plates (ThermoScientific) were imaged for 2 h with a time interval of 90 s using inverted Zeiss widefield microscope Observer.Z1 equipped with a 20× air objective. Analysis of HSPCs under stroma was performed in ImageJ by manual counting HSPCs recognized by phase contrast.

Supplementary Table S1. Primers and Probes Used for mRNA Quantification, Listed 5′ to 3′

Primers	*Fw*	*Rv*
TGFBI	ccctagtgagactttgaaccgtatc	ttcaagatgtggttgttcagcag
CCNA1	ggttcctttcatgtatgtctgttctg	gacaaactcgtctacttcaggaggata
CCNA2	tctagcgcagcagcagagg	gacatgctcatcatttacaggaaga
CCND1	ccatgcggaagatcgtcg	ggaagacctcctcctcgcac
CCND2	tccaaccctacatgcgcag	tcttcgcacttctgttcctcac
CDKN1b	agcaatgcgcaggaataagg	Tctgttggctcttttgttttgagt
GUS	gaaaatatgtggttggagagctcatt	Ccgagtgaagatccccttttta
Probes	*Sequence*	*Label*
TGFBI	cagtcatcagctacgagtg	5′FAM +3′TAMRA
GUS	ccagcactctcgtcggtgactgttca	5′FAM +3′TAMRA

MSC differentiation assay

For MSC lineage differentiation toward adipocytes and osteoblasts, StemPro Adipogenesis Differentiation Kit (A1007001; Gibco) and StemPro Osteogenesis Differentiation Kit (A1007201; Gibco) were used. MSCs of passage 3–4 were seeded at 1×10^4 cells/cm^2 and after 1 day, the medium was replaced by the differentiation medium, which was refreshed every 3–4 days. After 21 days, cells were fixed with methanol and analyzed for their lipid droplet content by means of Oil Red O staining and calcium deposits by means of Alizarin Red S staining. Total fluorescence intensity of Oil Red O staining was determined using inverted Zeiss widefield microscope Observer.Z1 (Ex 559–585; Em 600–690). Levels of calcific deposits were determined by total absorbance levels (ABS BP 480/9 nm) as measured by Infinite F200 pro plate reader (TECAN).

Statistical analysis

To statistically analyze the difference between two experimental groups, the student's *t*-test was performed; for multiple group comparisons, two-way Anova was used, *P*-values of ≤0.05 were considered significant.

Supplementary References

S1. Gammaitoni L, S Bruno, F Sanavio, M Gunetti, O Kollet, G Cavalloni, M Falda, F Fagioli, T Lapidot, M Aglietta and W Piacibello. (2003). Ex vivo expansion of human adult stem cells capable of primary and secondary hemopoietic reconstitution. Exp Hematol 31:261–270.

S2. Gammaitoni L, S Lucchi, S Bruno, M Tesio, M Gunetti, Y Pignochino, G Migliardi, L Lazzari, M Aglietta, P Rebulla and W Piacibello. (2006). Serial transplantations in non-obese diabetic/severe combined immunodeficiency mice of transduced human CD34+ cord blood cells: efficient oncoretroviral gene transfer and ex vivo expansion under serum-free conditions. Stem Cells 24:1201–1212.

S3. Maijenburg MW, WA Noort, M Kleijer, CJ Kompier, K Weijer, JD van Buul, CE van Der Schoot and C Voermans. (2010). Cell cycle and tissue of origin contribute to the migratory behaviour of human fetal and adult mesenchymal stromal cells. Br J Haematol 148:428–440.

S4. van Rijssel J, J Kroon, M Hoogenboezem, FP van Alphen, RJ de Jong, E Kostadinova, D Geerts, PL Hordijk and JD van Buul. (2012). The Rho-guanine nucleotide exchange factor Trio controls leukocyte transendothelial migration by promoting docking structure formation. Mol Biol Cell 23:2831–2844.

SUPPLEMENTARY MOVIE S1. Imaging of co-cultures reveals an increased fraction of HSPCs under stromal layer in *TGFBI* knockdown compared to control MSCs. Percentage of HSPCs present under the stroma was determined as a percentage of total HSPCs per field of view. Co-cultures were imaged on an inverted widefield microscope for 2 h, with a time interval of 90 s. A representative experiment is shown. Scalebar is 100 μm. *TGFBI*, transforming growth factor beta-induced gene H3; HSPC, hematopoietic stem and progenitor cell; MSC, mesenchymal stromal cell.
